# Seroprevalence and Risks Factors Associated with *Coxiella burnetii* Infection in Slaughterhouse Zebu Cattle (*Bos indicus*) from Northern Regions of Cameroon

**DOI:** 10.3390/epidemiologia3040033

**Published:** 2022-10-06

**Authors:** Camille Teitsa Zangue, Justin Kouamo, Ferdinand Ngoula, Ludovic Pépin M’bapté Tawali, Mathias Mba Talla, Lionnel Yvan Kantchouet Mbeba, Claude Landry Makuetamang Doumtsop, Bernard Viban Tangwa

**Affiliations:** 1 School of Veterinary Medicine and Sciences, University of Ngaoundere, Ngaoundere P.O. Box 454, Cameroon; 2 Faculty of Agronomy and Agricultural Sciences, Department of Zootechnics, University of Dschang, Dschang P.O. Box 96, Cameroon; 3 Regional Centre of the Institute of Agricultural Research for Development Wakwa, Ngaoundere P.O. Box 65, Cameroon

**Keywords:** *Coxiella burnetii*, slaughterhouse, zebu cattle, Cameroon, risks factors

## Abstract

A study was conducted to determine the seroprevalence and risks factors of *Coxiella burnetii* in zebu cattle from the northern regions of Cameroon. From a total of 2016 (1754 females and 262 males) sera sampled, 801, 762 and 453 were collected, respectively, from Adamawa, North and Far North, and screened for *Coxiella burnetii* using indirect enzyme-linked immunosorbent assay (iELISA). A total of 23.76% (479/2016) were serologically positive. The seroprevalence of Adamawa, North and Far North were 29.09% (233/801), 19.95% (152/762) and 20.75% (94/453); respectively. The seropositivity of male and female were 4.58% and 26.62%; respectively. Cattle from Adamawa region were more likely to have been exposed to *C. burnetii* than animals from Far North region (OR = 3.28; 95%CI: 1.13–7.85; *p* = 0.02). The Gudali breed was significantly more infected than Aku (OR =2.52; 95%CI: 1.06–5.99; *p* = 0.03), and animals aged of (6–9) years were 1.89 times more likely to have been infected to *C. burnetii* than young animals (*p* = 0.03). The seropositivity to this bacterium was significantly associated to pregnant cattle than non-pregnant (OR = 1.71; 95%CI: 1.01–2.90; *p* = 0.04). Female cattle were more likely to have been infected by *C. burnetii* than male and the rainy season were 1.66 more associated to this disease than dry season. The linear regression model indicated that *C. burnetii* seropositivity were positively correlated to the regions (0.09, CI: 0.04; 0.18; *p* = 0.007), age (0.01, CI: −0.01; 0.04; *p* = 0.02), sex (0.19, CI: 0.08–0.32, *p* = 0.001) and physiological status (0.11, CI: −0.04; 0.26; *p* = 0.006). This study revealed that *C. burnetii* infection is widespread among zebu cattle of Adamawa, North and Far North of Cameroon.

## 1. Introduction

Q fever is a zoonotic infectious disease caused by *Coxiella burnetii,* with a worldwide-distribution [1]. It is an emerging public health threat as it causes reproductive failures and production losses in domestic ruminants that are the primary reservoir [2]. This bacterium is relatively resistant to environmental conditions and can be transmitted from animal to animal or from animal to human by wind [3]. Animals can carry the infection for several years or lifelong, shedding the organism in various secretion and excreta, with increasing public health risk to cattle farmers, veterinarians, abattoir workers and consumers of animal products. The disease caused late-term abortion in ruminants, especially in sheep and goats [4]. This infection is often asymptomatic in human with a large polymorphism, but several symptoms can be observed at the acute form [5].

Many studies have reported the presence of *Coxiella burnetii* in several countries with varying prevalence’s [6]. In developing countries, the prevalence of infection with *C. burnetii* among domestic ruminants was estimated to be around 25.0% [7]. Several studies in cattle have shown a seroprevalence of 3.23% in domestic cattle of Southeastern Iran [8], 4–2.0% in Ulleung Island, Korea [9], 6.2% in Maigana and Birnin Gwari agro-ecological zone of Nigeria [10], 43.0% in Ecuador [6], 8.4% in Algeria [11], 23.5% in Ibarapa area of Nigeria [12], 18.0% in turkey [13] and 19.4% in South Africa [14]. The factors associated with a higher risk of exposure to *C. burnetii* are breed, herd size of cattle [12], veterinarians, abattoir and farm workers [14]. 

Little information is available regarding bovine *Coxiella burnetii* infections in Cameroon, though seroprevalences of 14.6% (CI 11.8–18.0%) in North West region and 12.4% (CI 9.6–15.9%) in Vina division of Adamawa have been reported [15]. The seroprevalence of *C. burnetii* in Adamawa, North and Far North is unknown, and most of the slaughter animals of these regions are females. Sampling in abattoir gives access to large number of animals from different localities, hense determining the prevalence of a disease on a large sample in a short time frame [16]. Estimating the *C. burnetii* seroprevalence and associated risk factors in zebu cattle of Adamawa, North and Far North regions of Cameroon could contribute to it control in animals and indirectly in humans.

## 2. Materials and Methods

### 2.1. Study Area 

**Figure 1:**
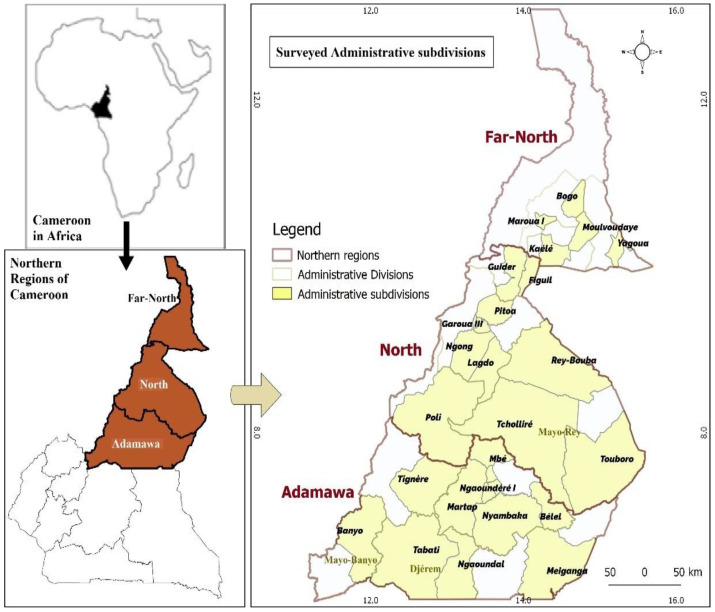
Map of study area.

The study was conducted in Northern (Adamawa, North and Far North) Regions of Cameroon (Figure 1 and Table 1).

### 2.2. Samples Size Determination

Serum samples were obtained from slaughterhouses of twenty-five localities in the Adamawa, North and Far North regions of Cameroon, from May 2021 to March 2022. The sample size was determined using the formula: N = (Z2 × P (1 − P))/d2 [18], where Z = 1.96 from normal distribution table, P = expected prevalence, d = desired precision- level. The conservative estimate of 39% prevalence reported in Adamawa region of Cameroon [19], 95% level of confidence and 3% absolute precision was used [20]. Accordingly, the estimated sample size of 938 animals was obtained. To avoid sample bias, 2016 cattle *Bos indicus* samples were collected, divided among regions based on the number of cattle slaughtered per day, after consensus from the veterinary inspection service and owner of animal. The average daily output of these slaughterhouses was 75.18 ± 12.99 (11.46–142.54), 66.21 ± 4.65 (7.42–92.87) and 64.44 ± 12.52 (3.98–147.40) in Adamawa, North and Far North; respectively.

### 2.3. Specimen Collect Procedure

About 10 mL of blood sample was collected from the jugular vein of each selected cattle in tubes, without additives, every thirty minutes depending on the rate of animal slaughter, and transported the same day on ice cooler to the laboratory, and processed. Each specimen was labeled with unique identification number. Serum was harvested same day by centrifuging at 4000 rpm for 20 min and stored at −20 °C for analysis. Each sample was properly labelled with relevant information such as the region, age, breed, body condition score, sex, season and physiological status (pregnant or none).

### 2.4. Laboratory Analysis and Interpretation 

All serum samples were assayed with Indirect Enzyme Linked Immunosorbent Assay (i-ELISA) from ID Screen^®^ Q fever Indirect Multi- Species kits (ID.vet, 310; rue Louis Pasteur (Grabels, France)) for the detection of antibodies against *C. burnetii*, which was carried out using Phase I and II purified antigens of *C. burnetii*. This ELISA has a sensitivity of 100% (CI 95%: 89.28–100%) and a specificity of 100% (CI 95%: 97.75–100%). The optical densities (OD) were measured at 450 nm in a micro-plate photometer. Interpretation of OD was according to the manufacturer’s instructions. The result for each sample was obtained as the percentage of the ratio between the sample Optical Density (OD) and positive control OD, according to the Substrate/Product percentage (S/P%), as given below.

$$\frac{s}{p}\%=\frac{\text{OD Sample}-\text{OD negative control}}{\text{OD positive control}-\text{OD negative control}}\times 100$$

Any sample with an S/P% ≤ 40% was considered seronegative; if the S/P% was between <40% and ≤50%, the result was considered doubtful, the S/P% between <50% and ≤80% was classified as seropositive, and Strong positive if the S/P% was >80%.

### 2.5. Statistical Analysis

The data were analysed using Statistical Package for Social Sciences (SPSS) version 23.0. Descriptive statistics were used to calculate the prevalence and summarized as frequencies. The logistic regression analysis was used to compare the proportions of detected sample positivity indifferent regions and among different animals to identify risk factors for *C. burnetii*. The differences were considered to be statistically significant when the resulting *p*-values were lower than 0.05 and 0.01. 

## 3. Results

### 3.1. Seroprevalence of Coxiella burnetii in Northern Regions

A total of 479 positive samples demonstrated the total seroprevalence as 23.76% (479/2016), and seroprevalence of Adamawa, North and Far North regions were 29.09% (233/801), 19.95% (152/762) and 20.75% (94/453); respectively. However, seroprevalence among the slaughterhouse is between 8.51–44.90%; 5.88–25.00% and 14.49–28.79% in Adamawa, North and Far North; respectively. The lowest and highest values were obtained in Poli (Faro division in North region) and Meiganga (Mbere division in Adamawa region); respectively (Table 2).

### 3.2. Risks Factors of Coxiella burnetii in Northern Regions of Cameroon

Cattle from Adamawa region were more likely to have been exposed to *C. burnetii* than animals from Far North region (OR = 3.28; 95%CI: 1.13–7.85; *p* = 0.02). The Gudali breed were significantly more infected than Aku breed (OR = 2.52; 95%CI: 1.06–5.99; *p* = 0.03). Animals aged of [6,7,8,9] years were 1.89 times more likely to have been infected to *C. burnetii* than young animals (*p* = 0.03). The seropositivity to this bacterium was significantly associated to pregnant cattle than non-pregnant (OR = 1.71; 95%CI: 1.01–2.90; *p* = 0.04). Female cattle were more likely to have been infected by *C. burnetii* than male and in the rainy season were 1.66 more associated to this disease than dry season (Table 3). These results indicated that *C. burnetii* seropositivity were positively and significantly correlated to the regions (0.09, CI: 0.04; 0.18; *p* = 0.007), age (0.01, CI: −0.01; 0.04; *p* = 0.02), sex (0.19, CI: 0.08–0.32, *p* = 0.001) and physiological status (0.11, CI: −0.04; 0.26; *p* = 0.006) (Table 4).

## 4. Discussion

These results have showed that *C. burnetii* is an infection which is present and represents a public health problem in the Cameroon. A seropositivity of 23.76% was obtained on tested animals in study areas. These findings are in agreement with study undertaken in nine slaughterhouse in Brazil which reported 23.8% (360/1515) serologically positive by Immunofluorescence assay [20], but higher than similar reported in South Western of Ethiopia (11.79%, CI: 7.63–17.17) [21], in Saudi Arabia (9.1%, CI: 6.7–12.1) [22], in Northwestern region of Spain (18.4% in cattle) [23], in North west region (14.6%, CI 11.8–18.0%) and Vina division of Adamawa (12.4%, CI 9.6–15.9%) of Cameroon [15], but lower than 39% in Adamawa region of Cameroon [19], 50.7% in Assiut, Egypt [24] and 43% in cattle from Ecuador [6]. This higher positivity on *C. burnetii* in Adamawa, North and Far North of Cameroon may be attributed to the extensive husbandry system, and the fact that all cattle of this study were local breeds in close contact with others species (sheep and goat) and wildlife. 

The results of this study indicates that the northern regions are positively associated to *C. burnetii* (*p* = 0.005) and cattle from the Adamawa region were 3.28 more likely to be seropositive than Far North region (*p* = 0.02). The Adamawa region of Cameroon is one of the main transit points of cattle because of the quality of its pasture and its geographical position between the northern and southern part of the country. It is a meeting place for many herds from neighboring countries, such as Nigeria, Chad, and Central African Republic, and even from the North and Far North regions of Cameroon, who come in transhumance. Likewise, the climatic conditions of this region, with average temperature of 21.8 °C and rainfall of 951.9 mm, favors the development of pest like ticks, in this environment [19]. 

There was no significant correlation between breed and the seropositivity to *C. burnetii* (*p* < 0.05), but Gudali were more likely to have this disease than Aku. In Cameroon, the Gudali breed in mainly raised in restricted areas of Adamawa region and this could increase their contact with the pathogens. Although the infection rate influence by these differences in breed is not clearly clarified. These results have showed that BCS is negatively associated to *C. burnetii* and heavy cattle presented higher prevalence than light. In this study, the age was positively and significantly associated to the infection and probability to *C. burnetii*; exposure of cattle increased with age. These results are in agreement with previous studies in Ethiopia [21] and Saudi Arabia [25]. The older the animal, the more likely its exposure to the pathogen infections at some point in his life [26], which is more marked with the extensive husbandry system practice in the study areas. Some authors reported that the predominant contagious route of the infections is being more horizontal transmission than vertical, and this may explain the higher prevalence in old animals [27]. *Coxiella burnetii* seroprevalence was higher in the rainy season than in the dry season. This could be due to the breeding system in the study areas, the animals are generally crossed in the dry season so that the birth takes place during the rainy season and the female can benefit from the quality grass in order to feed her calf. The onset of the rainy season leads to the development of ectoparasites and ticks in particular, which are vectors of *C. burnetii* [28].

The results of risk factor identification among animals showed that female cows were at significantly high risk of infection with *C. burnetii.* This result is comparable with those reported in the eastern region of the Kingdom of Saudi Arabia [22]. The causes of higher seroprevalence among females could be that *C. burnetii* has a high affinity for the mammary glands and uterus [29]. This study showed that physiological status was positively correlated to the presence of the *C. burnetii* and pregnant cattle were 1.71 more likely to be infected than non-pregnant (*p* < 0.05), with high seroprevalence in both two groups. Generally, the disease is asymptomatic in domestic ruminants and the exclusive clinical manifestation is abortion [30]. The most visible manifestations are observed in pregnant females, and depending on the virulence of the germ, it could have necrosis of the cotyledons leading to fetal anoxia, and therefore an abortion. If the bacteria are less virulent, it could result in the birth of a frail calf that is more susceptible to infection [31]. The presence of *C. burnetii* would cause a decrease in gestation hormone 17ß-estradiol and the levels of IgG PhI/II in late pregnancy, resulting in the loss of the fetus [32]. To further explicate the epizootiology of *C. burnetii* infection in Cameroon, the studies on the milk and vaginal samples, uterine-discharges, abortive materials and semen of cattle bulls along with identification of ticks for the presence of the organism are paramount important. This study has some limitations due to the non-investigation of the ticks as reservoirs of infection and lack of sequencing equipment for accurate diagnosis, however, the number of samples processed and the large study area revealed that zebu cattle are the reservoirs for Q fever in Cameroon. 

## 5. Conclusions

This study showed that *Coxiella burnetii* is widely spread among zebu cattle of the Adamawa, North and Far North regions of Cameroon, with seroprevalence of 29.09% (233/801), 19.95% (152/762) and 20.75% (94/453); respectively. Depending of the sex of the slaughtered animals, the seroprevalence of male and female was 4.58% and 26.62%; respectively. The main cattle risks factor identified were region, age, sex and physiological status of females (*p* < 0.05). Q fever is a neglected zoonotic disease in Cameroon and there are no control measures because the level of diagnosis is low, due to the country’s poverty, absence of animal vaccination and the high cost of disease detection techniques. Surveillance programs for humans and animals should be implemented to monitor cases of Q fever and establish preventive and control measures. The high prevalence obtained in this study shows that research should be done on the human risk groups, like cattle farmers veterinarians, abattoir workers and consumers of animal protein, and to determine the economic impact of this disease in the cattle industry of Cameroon. Hence, it necessitates the attention of the veterinary and public health authorities, using the one-health approach to control its occurrence.

## Figures and Tables

**Table 1:** Study area characteristics [17].

Characteristics	Regions		
	Adamawa	North	Far North
Latitude	5°–8° N	8°–10° N	10°–12° N
Longitude	11°–14° E	12°–14° E	14°–15° E
Surface Area (km^2^)	63,701	66,090	34,263
Climate	Sudano Guinean	Soudanese	Sudano sahelian
Mean annual temperature (°C)	22–25	21–34	25–35
Mean annual precipitation (mm)	900–1500	800–900	600–1000
Number of slaughtered animals in 2018 (Head)	110,495	76,677	62,598

mm—millimeters; km^2^—Square kilometer; °C—Celsius degree; N—North; E—East.

**Table 2:** Seroprevalence of *C. burnetii* for each slaughterhouse in northern regions of Cameroon (*n* = 2016).

Northern Regions	Divisions	Slaughterhouse	Samples Analyzed	Positive	Prevalence (%)
Adamawa	Djerem	Tibati	74	18	24.32
		Ngaoundal	65	14	21.54
		Total	139	32	23.02
	Faro et Deo	Tignere	47	4	8.51
	Mayo Banyo	Banyo	111	28	25.23
	Mbere	Meiganga	98	44	44.90
	Vina	Ngaoundere	201	74	36.82
		Belel	51	16	31.37
		Martap	44	6	13.64
		Nyambaka	48	11	22.92
		Mbe	62	18	29.03
		Total	406	125	30.78
	Total		801	233	29.09
North	Benoue	Garoua	144	29	20.14
		Ngong	62	12	19.35
		Lagdo	46	7	15.22
		Pitoa	54	13	24.07
		Total	306	61	19.93
	Faro	Poli	51	3	5.88
	Mayo louti	Figuil	71	16	22.54
		Guider	68	17	25.00
		Total	139	33	23.74
	Mayo Rey	Touboro	92	21	22.83
		Rey Bouba	111	26	23.42
		Tchollire	63	8	12.70
		Total	266	55	20.68
	Total		762	152	19.95
Far North	Diamare	Maroua	149	37	24.83
		Bogo	69	10	14.49
		Total	218	46	21.10
	Mayo Kani	Moulvoudaye	66	19	28.79
		Kaele	82	12	14.63
		Total	148	31	20.95
	Mayo Danay	Yagoua	87	16	18.39
	Total		453	94	20.75

%—percentage.

**Table 3:** Results of logistic regression analysis for risk factors identification for *C. burnetii* infection among slaughterhouse zebu cattle (*n* = 2016) in Northern Regions of Cameroon.

Variable		Number Tested	Number Positive	Prevalence (%)	*p* Value	OR	95% CI
Regions	Far North	453	94	20.75	Referent		
	Adamawa	801	233	29.09	0.02 *	3.28	1.13–7.85
	North	762	152	19.95	0.49	3.02	1.06–6.90
Breed	Aku	563	121	21.49	Referent		
	Bokolo	162	43	26.54	0.85	1.10	0.41–2.96
	Djafun	408	83	20.34	0.43	1.35	0.64–2.85
	Gudali	883	232	26.27	0.03 *	2.52	1.06–5.99
Age (years)	[3,4,5]	1074	175	14.62	Referent		
	[6,7,8,9]	672	235	34.97	0.03 *	1.89	1.06–3.35
	≥10	270	69	25.56	0.30	1.47	0.71–3.05
BCS	[1,2]	1481	178	12.02	Referent		
	3	402	232	57.71	0.64	0.91	0.36–2.33
	[4,5]	133	69	51.88	0.84	1.07	0.42–2.41
Physiological status (female)	Non pregnant	1497	358	23.91	Referent		
	Pregnant	257	109	42.41	0.04 *	1.71	1.01–2.90
Sex	Male	262	12	4.58	Referent		
	Female	1754	467	26.62	0.002 *	1.92	0.61–3.81
Season	Dry	1293	258	19.95	Referent		
	Rainy	723	221	30.57	0.13	1.66	0.69–4.01

Values in a column with “*” differ significantly at *p* < 0.05; CI—confidence interval; OR—Odds ratio, %—percentage.

**Table 4:** Model analysis of with *C. burnetii* seropositivity among zebu cattle sampled from slaughterhouse (*n* = 2016) in the northern regions of Cameroon (Adamawa, North and Far North).

Factors	*C. burnetii* (95% CI)	*p* Value
Regions	0.08 (0.01; 0.17)	0.005 **
Breed	0.04 (−0.02; 0.11)	0.09
Age (years)	0.03 (−0.03; 0.11)	0.04 *
BCS	−0.01 (−0.06; 0.04)	0.46
Physiological status (female)	0.11 (−0.05; 0.22)	0.006 **
Sex	0.19 (0.08–0.32)	0.001 **
Season	−0.14 (−0.33–0.05)	0.16
Constant	1.19 (0.73; 1.64)	0.000 **
*N* = 2016	R^2^ = 0.03	

** *p* < 0.01; * *p* < 0.05; CI: Confidence Interval.

## Data Availability

The data presented in this article are not available publicly due to ethical reasons and are available from the corresponding author upon reasonable request.
